# Physicians’ Perceptions and Experiences Regarding Leadership: A Link Between Beliefs and Identity Formation

**DOI:** 10.2147/JHL.S464289

**Published:** 2024-07-01

**Authors:** Robin Lüchinger, Marie-Claude Audétat, Nadia M Bajwa, Anne-Claire Bréchet-Bachmann, Hélène Richard-Lepouriel, Melissa Dominicé Dao, Noëlle Junod Perron

**Affiliations:** 1 Unit of Research and Development in Medical Education, Faculty of Medicine, University of Geneva, Geneva, Switzerland; 2 University Institute of Family and Child Medicine, Faculty of Medicine, University of Geneva, Geneva, Switzerland; 3 Department of Women, Children and Adolescents, University Hospitals of Geneva, Geneva, Switzerland; 4 Department of Primary Care Medicine, University Hospitals of Geneva, Geneva, Switzerland; 5 Department of Psychiatry, University Hospitals of Geneva, Geneva, Switzerland; 6 Medical Directorate, University Hospitals of Geneva, Geneva, Switzerland

**Keywords:** leadership, physicians, identity formation, beliefs, perceptions, qualitative

## Abstract

**Introduction:**

Despite the development of national recommendations and training programs for effective leadership, junior and senior medical leaders often find themselves ill-prepared to take on these new responsibilities. This study aimed to explore physicians’ perceptions, feelings, and beliefs regarding leadership and to provide recommendations regarding appropriate training and institutional post-training support.

**Methods:**

We conducted a qualitative study at the Geneva University Hospitals. A purposeful sample of residents (R), fellows (F), attending physicians (A), and chairpersons (CP) were invited to participate in focus groups (or semi-structured interviews) between April and June 2021. We investigated their understanding of leadership, self-perception as leaders, difficulties, and paths to improvement in their leadership skills. Focus groups were transcribed verbatim and analyzed both inductively and deductively using Fishbein’s model of behavior prediction and Irby’s professional identity formation framework.

**Results:**

We conducted ten focus groups (R=3; F=4, A=2, and CP=1) and one interview (CP). Physicians expressed poor self-efficacy at all hierarchical levels: feelings of insecurity and confusion (R and F), frustration (A), and feeling stuck between divisional and institutional governance (CP). Such negative feelings were nurtured by personal beliefs with an intuitive and idealized representation of leadership. Beliefs focused more on personal characteristics rather than on skills, processes, or perceived institutional norms. Unclear expectations regarding physicians’ role as leaders, overemphasis on academic achievement, and silo professional organizations fueled their feelings. Participants reported developing their leadership through trial and error, observing role models, and turning to personal resources rather than formal training.

**Conclusion:**

Our results show that physicians’ leadership skills are still mainly acquired intuitively and that institutional norms do not encourage clarification of leadership roles and processes. Physician training in leadership skills, together with more explicit and clear institutional processes, may help to improve physicians’ self-efficacy and develop their identity as leaders.

## Introduction

Effective medical leadership is essential to the success of any healthcare organization since physician engagement in the leadership of health systems has been shown to improve patient care and organizational performance,^1–3^ staff well-being,^3^ and patient outcomes.^4^ Medical leadership is usually described as a combination of both medical and managerial work^5^ for which physicians are expected to demonstrate both leadership and management skills.^6^ Management implies handling complexity, referring to resources’ organization and optimization to achieve tasks and goals.^7^ Leadership is about dealing with change, requiring the ability to motivate, inspire, and empower others to achieve objectives.^8^,^9^ Although leadership and management are complementary constructs, we will refer to them as a singular concept (L&M) in this article since physicians are expected to perform both leadership and management tasks in healthcare institutions.

As highlighted by the NHS leadership framework, physicians need L&M competences to become more actively engaged in planning, delivering, and transforming healthcare services.^10^ Such competences cover several leadership dimensions, such as personal qualities, unit management, interprofessional collaboration, and organization leading. However, physicians are often parachuted into leadership and management positions based on their clinical expertise and often lack the skills necessary for such organizational roles.^11–14^ In response to such needed skills, several training programs in medical leadership have been developed worldwide and in Switzerland.^15–19^ However, several studies have highlighted physicians’ need for attendance in such programs.^20–22^

Reasons for non-attendance have been explored and include external factors such as lack of protected time, lack of institutional support or perceived value by the organization.^23^ Personal factors are related to physicians’ skepticism about their need to train in L&M skills and an inherent tension between managing care and providing care.^23^ Endorsement of leadership may be facilitated by the acquisition of skills or the development of an identity as a leader.

Some theories aim to explain the relationship between attitudes and behaviors within human action. They are mainly used to predict how individuals will behave based on their pre-existing attitudes and behavioral intention, these attitudes and intentions being influenced by beliefs regarding self and organization norms.^24^ For example, demonstrating leadership skills is most likely to occur if the intention to perform that behavior is assertive and is aligned with institutional norms and if the person possesses the required skills and feels legitimate to use such skills. Medical leadership may also imply an identity shift for the physician.^25^ Several identity theories grounded in sociology, psychology, and philosophy have explored the concept of identity and identity formation.^26^ Whatever lens is adopted, there is agreement that professional identity can be defined as the combination of attitudes, beliefs, motives, values, and experiences through which people define themselves in a professional role.^27^ It is a process of adaptive development that occurs simultaneously at two levels: at the individual level, relating to the psychological development of the person, and at the collective level, which involves the socialization of the person into appropriate roles and forms of participation in community work.^28^ While the links between leadership identity development and demonstration of leadership skills and performance have been explored among physicians having adopted formalized leadership positions inside health institutions,^3^,^29^ less is known about how these concepts interconnect among physicians in training or to those recently appointed in middle leadership positions. This is of importance since such groups of physicians are often unaware of their role and skills as leaders in clinical practice and tend to consider that only physicians with designated leadership roles and involved in institutional governance are legitimate leaders. However, they play a pivotal role in the operation of services and institutions and carry out L&M tasks on a daily basis.^30^ This study aimed to explore physicians’ perceptions, feelings, and beliefs regarding leadership and management among residents, fellows, attending physicians, and chairpersons in order to better understand the facilitating and impeding factors influencing their performance and self-efficacy as leaders at different organizational levels. We were particularly interested in the perceptions and beliefs of physicians in training or physicians in middle L&M positions for the abovementioned reasons. The overall aim is to provide recommendations regarding appropriate training and institutional post-training support in order to help physicians feel like leaders and use L&M skills at all organizational levels.

## Method

### Design, Setting, and Participants

We conducted a qualitative study using focus groups at the Geneva University Hospitals in Switzerland between October 2020 and April 2021.

### Participants

Participants were residents, fellows, attending physicians, and chairpersons of various hospital departments who were selected according to a purposeful sampling approach. A sample of physicians who answered an institutional online survey in 2020 about their self-perceived competencies and training needs regarding leadership and management and expressed interest in such issues were invited to take part in this study (n= 99).^21^ The sampling took into account the medical disciplines and the level of hierarchy. We also invited a random list of physicians from different disciplines and levels of hierarchy who did not participate in the survey in order to include physicians who had not previously expressed an interest in leadership (n=30). Potential participants were sent an email invitation explaining the goal of the research as well as information and consent forms (Appendix 1).

### Data Collection

We conducted separate focus groups with physicians of four hierarchical levels (residents, fellows, attending physicians, and chairpersons). We chose focus groups to explore the knowledge and experience in leadership and management from different perspectives at the hierarchical levels. This method allows physicians to exchange freely among their peers from different departments and to elicit a wide range of ideas and opinions on a well-defined topic.^31^ We also included a semi-structured interview with a chairperson who was willing to take part but was not available for the focus group time slot. The focus group guide included questions about participants’ perceptions of L&M, barriers and facilitators regarding L&M in the field, and how to develop L&M skills. We adapted the guide for chairpersons to integrate questions about their perceptions and experience regarding the L&M roles of subordinate physicians (Appendix 2).

Each focus group lasted 75–90 min and took place online using the *Zoom* video-conferencing platform (version 5.4.9) because of the restrictions linked to the COVID-19 pandemic. Focus groups were conducted by a pair of facilitators (among RL, NJP, MCA, and HRL) who had no past or current hierarchy link with the participants in order to ensure that participants felt free to express their views. Debriefing discussions between facilitators at the end of the focus group were audiotaped and transcribed immediately after each session. Focus groups were transcribed ad verbatim.

### Analysis

**Figure 1:**
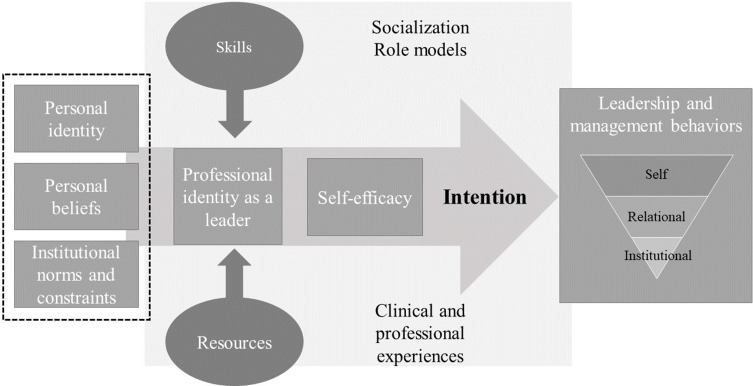
Integrated model of professional identity and predictive behaviour for medical leadership.

We conducted a thematic analysis through several phases.^32^ In the first phase, all investigators read all focus groups, noting keywords and phrases. After discussion, an initial list of codes was developed using a deductive approach based on elements of Fishbein’s model of predictive behavior^24^ and Cruess’s model of identity formation and socialization^33^ (Figure 1) and further enriched through discussions. The integrated model of Fishbein^24^ suggests three primary determinants of intention: behavioral beliefs, normative beliefs, and efficacy beliefs. Behavioral beliefs are the person’s overall feelings of favourableness or unfavourableness toward performing the behavior. Normative beliefs include both perceptions of what others think one should do as well as perceptions of what others are doing. Efficacy beliefs are one’s beliefs that one can perform the behavior even under challenging circumstances. The relative importance of these three psychosocial variables as determinants of intention will depend upon both the behavior and the population being considered. The model of medical professional identity formation and socialization includes multiple factors within and outside of the medical educational system that affect the formation of an individual’s professional identity.^33^,^34^ These factors include the existing personal identities nurtured by friends and family, and the professional identities supported by role models/mentors, clinical experiences, and formal teaching and rituals. The socialization process occurs both consciously and unconsciously through role models, mentors, and clinical and non-clinical experiences. Individuals tend to react to those different factors in their own way.

The final coding scheme included themes such as personal beliefs, perceived institutional norms, self-efficacy, emotions, tasks, skills, learning, professional identity, and dissonances/dilemmas. A smaller team (NJP, RL) individually hand-coded three transcripts, expanding on the initial thematic list where necessary. Any differences in the way themes were identified and coded were discussed. They were cross-checked by the larger group, and as they required only minor changes, NJP coded the remaining transcripts using Atlas ti.^35^ Once the coding was finished, the investigators met regularly to read and analyze the codes related to each theme and discussed similarities and differences according to participants’ hierarchical level. We are confident that saturation was reached after ten focus groups, as we were able to identify participants’ main perspectives on leadership and management issues. All translations of participants’ utterances are the authors’ (NMB and MDD are native speakers).

## Results

Fifty-six physicians participated (n=43 from the survey sample and n=13 from the non-survey sample). We conducted ten focus groups and one individual interview between April and July 2021. Participants were residents (25%), fellows (52%), attending physicians (16%) and chairpersons (7%). They represented nine medical specialties, mainly internal medicine (23%), psychiatry (23%), rehabilitation (12%), surgery (12%) and pediatrics (11%). The distribution of gender and medical disciplines is indicated in Table 1.

**Table 1:** Participants’ Gender and Medical Discipline Distribution

	Residents 3 Focus Groups		Fellows 4 Focus Groups		Attending Physicians 2 Focus Groups		Chairpersons 1 Focus Group and 1 Interview		Total
	F	M	F	M	F	M	F	M	
Psychiatry	4	3	3	1	1				13
General internal Medicine	1	1	4	4	1		1	1	13
Rehabilitation medicine	1		2	1	1	1		1	7
Surgery			1	4		1		1	7
Paediatrics	1		4	1					6
Gynaeco-obstetrics	3		1						4
Oncology				1	1				2
Cardiology						2			2
Anaesthesia				1		1			2
Total	14		29		9		4		56

**Abbreviations**: F, female; M, male.

In the following paragraphs, we will describe first the L&M tasks reported by the participants and the feelings of self-efficacy related to these tasks. We will then report the personal and institutional perceived norms that influenced participants’ feelings of self-efficacy as well as the coping strategies participants developed to face or overcome L&M challenges and develop both skills and professional identity of leader/manager. The main findings are summarized in Table 2.

**Table 2:** Main Findings Regarding Participants’ Tasks, Feelings of Self-Efficacy, Personal Beliefs, Perceived Institutional Norms and Coping Mechanisms Regarding L&M

	Tasks	Feelings	Beliefs	Perceived Norms	Coping Mechanisms
			Independent from the Hierarchical Level		
Residents	Management of self, time and team	Confusion, suffering	Physician’s main identity and role is to care	Unclear institutional expectations	Trial and error
Fellows	Clinical and team management			Institutional emphasis on academic more than management achievement	Role models (good and poor)
Attending physicians	Team and institutional project management Career development plans	Frustration, irritation, and bitterness	Vague and idealized image of a good leader/manager		Reliance on other members of the team
Chairpersons	Institutional strategies, innovation Career development plans	Institutional loyalty, feeling stuck between a rock and a hard place		Institutional organization in silo while transversal team working	External resources: reading, exchange with people working in other fields

### Perceived Tasks Regarding Leadership and Management

Physicians talked about their leadership and management tasks in relatively scattered ways. However, according to the hierarchical level, participants tended to describe different tasks, moving from managing themselves to managing the institution (Table 2).

Residents placed particular emphasis on self-, time, and team management. They were concerned about how to conciliate clinical, training, and academic activities within their working time. Residents working in psychiatric wards were more prone to report team management activities, including interprofessional coordination and task delegation to nurses and psychologists.

In terms of management, I’d say maybe in terms of the micro-management we do ourselves. In other words, how much time we devote to seeing patients, making telephone calls, talking to our bosses, going to supervision, psychotherapy, administrative work, research, and that’s not always easy either. (R 14.14-1:7)

Chief residents mainly described managing clinical activities inside a unit and putting much effort into empowering team members inside their unit, members being mainly residents and nurses.

We’re a bit like small business managers, having to manage trainees and interns. Interns who don’t have the same levels, the same expectations. And you have to improvise a little at first when you don’t necessarily know how. (F 12.12-5:6)

Attending physicians considered supporting the professional development of their medical team, including continuing education and career plans, a main task, along with managing daily clinical activities. In addition, they reported being responsible for project management tasks such as implementing collaborative practices, IT tools, or administrative processes.

In other words, it’s all about day-to-day management, with people working on a specific problem. For example, how do you organize the operating room for surgeons? How do we organize a consultation? […] And then there are the more strategic aspects, such as: Where will our department be in 5 years’ time? What are the department’s main strategic lines? (A 23.25-10-12)

Not surprisingly, the chairpersons mentioned tasks such as strategic planning and innovation in their services or departments, managing institutional projects or delegating them to subordinates, managing teams, and interpersonal conflicts.

It’s the organization of the service and the support for the employee, the implementation of training plans. It’s the whole organization of the department, which means we have to deal with people’s day-to-day work. And then there’s conflict management, team management, project implementation, project support, the operational side that has to be coordinated with the strategic side, it’s a bit of all that. (CP 16.16-11:8)

Physicians of higher hierarchical levels felt that their professional progression was associated with additional tasks without reducing or canceling their previous tasks. This resulted in an accumulation of various tasks that were experienced as burdensome.

There’s also something we need to think about, because we’re doctors first and foremost, and we have quite a wide and varied remit. And it’s true that every time we can load the mule up a bit more, we load it up. And as a result, it’s inevitably to the detriment of other tasks that might more logically fall to us. (A 21.4-7:73)

### Self-Efficacy and Task-Related Feelings

Physicians tended to express negative perceptions, resentment, and poor self-efficacy regarding their leadership and management tasks. Both residents and fellows felt confused regarding the tasks they were expected to perform and described that the process of learning how to develop L&M skills was painful and slow, often at the expense of the team and the patients.

Then, at the end of my three years, with all my disastrous experiences, things were starting to come together. But I thought it was a great, great, great pity that it had taken me three years to achieve something harmonious. During those three years, I find that the patient is enduring it. (R 1.7-4:5)

Attending physicians tended to express frustration, irritation, or bitterness regarding their role and tasks as leaders or managers. They indicated several reasons, such as a lack of training and information on how to carry out their tasks and a lack of knowledge of the language and processes related to such tasks.

The first thing that comes to mind is that the medical profession is not very well trained in this area. That there’s a huge gap, that there’s a kind of naivety about how things work. I had to take private lessons from the vice-director, who explained to me how to manage a project.there’s a naivety and a lack of understanding of the language of HR processes! We really need to be better trained in this, in the posts of attending. (A 5.5-10:12)

The chairpersons felt more comfortable in endorsing a role and performing L&M tasks. They described their role as being a buffer between higher institutional authorities and their service and protecting their staff from institutional pressures and paradoxical injunctions. Although most participants from all hierarchical levels highlighted the feeling of being between a rock and a hard place between higher governance and their team, this was especially expressed by chairpersons.

But that’s also why, and I think this is also a managerial challenge, it’s to manage all these frustrations on a daily basis but not to let them show. Because we also have a duty of loyalty to the institution. And that’s how, in our role, we manage all that, but without passing on this frustration to our staff. (CP 18.5-11:23)

Participants linked such negative reactions towards L&M to the fact that they felt ill-prepared to endorse such roles and had little or no training in this field, regardless of their level of hierarchy or clinical experience.

So, all of a sudden, I’m really alone. And that’s the difficulty. And now, when I’m the manager, we find ourselves too alone, in situations where we’re the ones taking responsibility. So, since there’s no training, everyone does things their own way. (F 29.6-2:26)

### Personal and Normative Beliefs

As reported below, participants experienced dissonance between the reality of their job, personal identity, beliefs, and perceived institutional norms and constraints that prevented them from considering themselves as effective and legitimate leaders.

### Personal Identity and Beliefs

Several participants from different hierarchical levels did not consider management tasks to be part of their role, which they believed should be focused mainly on patient care and clinical supervision.

But it’s true that I saw my role as being purely one of passing on knowledge to the residents, basically, with the management of the unit. In the end, it’s all the aspects that aren’t necessarily directly linked to medical activity, but which are more: The management of potential absences, of difficulties, perhaps screening for burn-out. (R 1.4-5:23)

There are relocation agencies who are there to talk to people and who are there to manage conflicts, so I’m also wondering whether we shouldn’t include the contribution of the outside world in this discussion. we should include the fact that we can outsource certain aspects of human resources management which may not necessarily be essential for a doctor to do. (A 21.4-7:73)

Several participants had a rather vague and idealized image of what a good and competent leader/manager is, and they remained rather general in mentioning the skills and attitudes related to such a role.

I also think that good leadership or management can only be sincere. And good leadership really has to inspire people, employees, to get involved, to give of themselves and then, like that, it works so that things also run smoothly when the head isn’t there. (F 27.4-8:38)

I think the ideal is an amalgam of theoretical knowledge, pragmatic tools. and then everything philosophical as well. For example, a philosophical criterion would be warmth, being friendly to people. (A 21.4-7:41)

They struggled to provide concrete examples of managerial and leadership practices in their own work setting and tended to oppose the “charismatic and inspiring” leader to the “controlling” manager handling work processes. Some participants shared the idea that L&M skills were somewhat innate.

I work with examples and models. And so, I have some models in mind, people who are really competent, who have a lot of quality in managing teams. But, as I said, these people have a natural talent. (F 41.41-3:19)

### Normative Beliefs and Institutional Norms

Many participants highlighted the fact that institutional expectations regarding their roles as leaders were unclear and implicit and sometimes confusing, whatever their hierarchical level.

Personally, I think that there are some fairly clear roles expected of managers. Managing the flow of patients, the waiting room, emergencies, the different emergency staff. And then there are less clearly defined areas where the expectations of my superiors or my own expectations of my team are not always formulated.for example, when there are medical errors or complaints or angry parents, it’s true that we quickly go looking for the manager or leader to clarify these situations. Sometimes it shows that everyone’s roles aren’t necessarily clear. (F 29.6-2:11)

They felt that the development of the needed L&M skills was left to personal initiatives as the institution itself did not value leadership as an identity that physicians should develop throughout their career through training and acquisition of skills.

But, unfortunately, I think there’s a lot of this idea that either you’re good or you’re not. And it’s not seen as a skill that you have to acquire anyway. Nobody will ever say: ”He’s no good, he doesn’t know how to intubate, so he doesn’t intubate”. So I don’t know, I don’t understand why there’s so much reluctance. It’s really the idea that it’s not part of our job, in fact. (F 29.6-3:19)

According to participants, this translated into a lack of institutional training dedicated to management inside regular structural medical training sessions, high emphasis on research/academic achievement for career development, promotions without any consideration regarding leadership skills and emotional intelligence, and finally lack of recognition and autonomy for executive roles for middle management.

The problem that this poses at managerial level is that it poses a problem of resources and legitimacy. In other words, the person who unofficially manages the structure, which is not yet a unit, does not have the resources to manage that unit.So you can’t steer and manage an entity that doesn’t appear in the steering tools because it’s not a unit. (A 6.5-10:20)

Some participants perceived that the lack of clear expectations regarding medical leadership and lack of regulation regarding task and power distribution within a team coexisted with a tendency to burden physicians with an increasing number of tasks that should be delegated to others, with a high risk of exhaustion and burnout-out.

That’s the definition of a doctor. No, but it’s true. anything that doesn’t fall within the remit of a particular category of worker, that’s the doctor. Anything that involves looking after patients, of course, is the job of the person who feels responsible for things. It’s a bit strange. It’s a bit caricatured and exaggerated, but we often get that impression. I can see my colleague nodding. (F 29.6-3:28)

A final element mentioned was, on the one hand, the institutional organization with hierarchy in a silo and, on the other hand, the transversal team working. Participants described this contrast as highly dysfunctional and as a barrier to effective team management.

The [University Hospital] is organized by department, with silos between the different departments. As the doctor in charge of an interdisciplinary consultation. And so, when I take managerial decisions, in relation to this unit, with a medico-economic, clinical and operational rationale, well, things get stuck with the silos that are intra-institutional. (A 6.5-10:4)

### Coping Strategies and Self-Efficacy Development

Participants described several strategies to cope with or to overcome such challenges. The first mentioned was learning L&M skills through trial and error, implying an uncomfortable position by lack of knowledge and possible impairment of quality of care and patient security.

For me, it’s a skill that’s expected once you get up the hierarchy. It’s not really taught to us. And I have the impression that it’s very much a matter of improvisation and personal know-how. (F 17.17-2:3)

However, the experience gained through learning “on the job”, although difficult, helped them become more comfortable with both their role and L&M skills.

When you’re a resident, you survive managing the patients in your care. When you’re a fellow, you survive your responsibilities for managing interns. And then, little by little, as you progress, you survive other responsibilities, or management responsibilities, and things happen, all in all, quite naturally, with time and experience of situations you’ve lived through. (F 72.74-3:33)

Participants also reported being guided by good or poor role models to which they had been previously exposed to during their training or working experience. The mix of good and not so good role models coupled with unclear expectations, made learning of L&M skills difficult.

I also based myself on people that I didn’t want to be like, that I didn’t want to repeat the same thing. I saw interns who were also suffering from this, from this lack of framework. Then we thought, how can we do this? What adjustments can we make? What could be useful? (R 47.49-9:15)

Very few participants considered their head of service to be particularly inspiring through the demonstration of effective and constructive leadership behaviors and the creation of a safe and stimulating working environment.

So once again, I think it’s special because our boss has been trained in leadership and management. And that transpires in his vision, in his practice and in the environment he has created in the department. (F 82.82-2:28)

Support from members of the care team was identified by younger participants as an important resource for developing skills as a leader and manager.

Because I’ve just started, and we’ve been left behind. There are lots of chiefs and deputies, and they’re very nice, but they’re also overworked, so you have to get going. My resource has really been the nursing team. So that’s why I’ve become convinced of the importance of this horizontal approach to decision-making. (R 7.4-4:21)

As an additional source of understanding of their role, some participants reported having relied upon external resources to improve their understanding of their role as leaders, such as informal discussions with relatives working as managers in other fields, reading books, or reviews about leadership. Others sought assistance or help from human resources inside the institution to learn the vocabulary and the structural processes involved in institutional project management.

And then, with my colleagues too, who have other backgrounds, in business or HR or management, I find it very rich. Then, I have not done much, but I have sometimes done coaching sessions. After that, I have always read books about other things too. (F 29.6–2:33)

## Discussion

The aim of this study was to explore Swiss physicians’ perceptions regarding L&M through the lenses of both behavioral prediction and professional identity development models, as illustrated in Figure 1. We showed that as physicians achieved higher hierarchical levels, leadership tasks moved from self-oriented to more team- and institutional-oriented. They all expressed poor self-efficacy regarding their L&M task at all hierarchical levels, with feelings of insecurity and confusion among residents, frustration among fellows, and attendings and chairpersons feeling stuck between department and institutional governance. Several factors, such as personal identity, beliefs, and perceived norms and constraints, prevented them from considering themselves as compelling and legitimate leaders. They all reported having learned how to become a leader through trial and error, observation of poor or sometimes good role models, and using personal resources more than formal training.

The shift from managing oneself to managing teams and institutions as physicians endorse higher positions and responsibilities is not surprising and aligns with the stages described in Kegan’s developmental theories of identity formation.^36^ Residents, when relating to leadership identity, are often more focused on their own needs and interests (finding a balance between clinical, training, and academic activities) than on relational or team issues. Fellows are able to view multiple perspectives simultaneously and are oriented towards teamwork (managing clinical teams involving residents and nursing staff). Chairpersons and attendings to some extent assume their role (leading and managing projects or services, planning careers for their team members, etc.) and understand and manage relationships in terms of different values and expectations.^33^ We showed that the challenges related to the L&M tasks were specific and varied according to the hierarchical level. These findings are in accordance with the NHS framework for medical leadership.^10^ However, successful medical leaders, whatever their hierarchical position, must not only possess appropriate competencies but also gain confidence in their ability to control events in order to achieve the desired goal-related tasks.^37^

Emotions related to L&M work were mainly negative, regardless of physicians’ hierarchical position. Similarly to prior studies conducted among formalized medical managers and leaders, participants described conflicting feelings and experienced difficulties in reconciling their role as health professionals with their role as leaders and managers.^38^ We showed that several elements related to personal identity and beliefs, as well as perceived institutional norms and constraints, conflicted with the harmonious development of their identity as leaders and nurtured such discomfort. In this study, residents, fellows, and attending physicians had an idealized vision of leaders and emphasized leaders’ personal characteristics more than L&M knowledge, best practices, or processes. This idealization may have conveyed the belief that leadership is innate and does not require training, hampering professional identity development as leaders. Indeed, physicians may experience difficulties in recognizing the abilities and expertise needed to undertake L&M positions.^23^ Adjusted expectations or a more realistic view of the requirements to perform as a leader reduce the intensity of negative emotions linked to disappointment.^39^,^40^ This could explain why most physicians reported rather negative feelings regarding their own leadership. A recent review showed that most formal leadership development programs help physicians gain knowledge of leadership roles or responsibilities, including skills in strategic planning, change management, and critical thinking, and improve communication skills as well as self-confidence.^41^ Moreover, such development programs also improved physicians’ understanding of broader processes, such as system thinking and managerial processes used to support change and improve patient outcomes.^42^ These findings indicate that L&M can be largely taught and donot only rely on personal (or innate) characteristics.

We also showed that unclear institutional expectations, overemphasis on academic achievement at the expense of L&M skills development, and the professional silo organization impacted negatively on the development of L&M skills and identity, especially among fellows and attending physicians. These findings highlight the importance of the workplace in influencing physicians’ professional identity as leaders and match prior findings.^43^ Physicians are often promoted to leadership positions based on their academic achievements with less emphasis on their L&M skills and experience,^44^ the assumption being that good clinical skills will compensate for flaws in leadership skills. However, this kind of compensation appears detrimental to leadership identity formation.^3^ The silo organization of healthcare institutions, with nurses being led by nurse leaders and physicians by physician leaders, brings an additional challenge when clinical work is performed interprofessionally with health professionals sharing different habits and values.^45^ This may negatively impact their ability to lead or collaborate,^46^ especially if such interprofessional collaboration is not supported by the institutional environment by clarifying roles and identity boundaries. Consequently, this may lead to frustration and stress.

We also found that physicians (especially fellows and attending physicians) tend to value clinical work to the detriment of L&M work and complain about the accumulation of tasks resulting from the endorsement of their role as leaders. As they move higher in the hierarchy, physicians prefer to give priority to providing direct patient care because patient care is an inherent part of their identity.^25^,^26^ Another reason reported in the literature is that physicians want to keep their clinical credibility while endorsing higher positions to maintain other physicians’ respect and followership.^42^ For such hybrid leaders who tend to emphasize their professional identity as a physician more than as a leader or manager,^43^ carrying out leadership work has been described as difficult because patient work often takes priority over everything else.^44^ They also see L&M work as an extension rather than an integration of such tasks into their professional role.^45^ This attitude, which can lead to feelings of frustration and overload, may be distinguished from other health professionals, such as nurses, who more easily adapt to administrative positions and see a managerial career as an alternative career to patient care.^46^,^47^

We highlighted that physicians, whatever the hierarchical position, developed their own coping strategies, such as on-the-job learning (becoming from performing) and observing (role modeling). From an individualist perspective, practicing and observing, together with reflective responses to experiences, are key factors in the process of identity formation.^43^,^47^,^48^ However, professional identity development as a leader is also dependent on how the institution supports the role of L&M.^47^ The fact that participants often solicited additional resources such as readings or discussions with professional managers outside the organization suggests that learning was largely implicit and opportunistic and indicates that the institution’s culture did not fully support formal learning of L&M nor set up clear expectations regarding medical leadership.

Finally, poor self-efficacy may be related to physicians’ type of personality, although this theme did not emerge from our data. Physicians are often described as independent people valuing autonomous decision-making and personal achievements while aiming to improve their own performance.^49^ This means that physicians think in terms of individuals^50^ while, as leaders, they need to collaborate with and even manage other healthcare resources and professionals. Working with interprofessional teams can be challenging and frustrating since such interactions may weaken their position and authority,^51^ especially if physicians tend to adopt an authoritative leadership style.^52^

## Recommendations

In order to sustain the development of professional identity as a leader or manager, and because our study shows that these needs appear early, training should start early during medical education in order to link this L&M dimension to the physicians’ identity together with patient care and teaching. In this perspective, Stoller^49^ suggested the development of self-awareness and emotional intelligence at an early stage and more relational/social competencies regarding L&M at later stages.

Leadership development programs in postgraduate or continuous training have been shown to be effective:^41^,^42^,^53^ participants develop knowledge and skills that will help them gain confidence in the role of L&M, supporting the distinction with the idea of natural talent or personal qualities alone. It can also be considered as an identity workspace where participants are stimulated to deconstruct and reconstruct professional identities through interactive and collective discussions as well as through self-reflection.^54^ However, to be attended, such programs need to be attractive and based on participants’ specific needs. For physicians in training or recently appointed in middle L&M positions, expanding faculty development programs initially designed for teaching skills to L&M skills training may help them coherently articulate and enrich their different professional identities as clinicians, teachers, and leaders. It may also be the opportunity to make the link between quality of care, effective teaching, and team management and improve their self-efficacy in these different fields.^53^ Physicians trained in L&M are more likely to become inspiring leaders using effective skills and explicit processes. Rewarding effective medical leaders is another way to change physicians’ mentality and may help them to consider L&M to be part of their professional identity.^18^,^55^

Adding coaching in the workplace will further bolster physicians’ ability to generate valuable L&M strategies and may support the enculturation process of endorsing leadership tasks and roles within the institution. Coaching may be beneficial in understanding, negotiating, and/or overcoming experiences of dissonance as physicians move on to new L&M positions and discover new roles and tasks. It highlights the fact that the institution itself has a responsibility to provide resources and support physicians in developing leadership roles and responsibilities.^44^ However, training and coaching activities should be aligned with clear institutional goals and strategies.^56^ This requires to ensuring the appropriateness of institutional goals for the missions entrusted to physicians, and also communicating and understanding these goals.

## Limitations

The study has some weaknesses. Given the differences in healthcare system organizations and the fact that this study took place in a single French-speaking University Hospital in Switzerland, our findings may not be applicable to other contexts. However, similar problems related to medical leadership have been reported in different countries.^15^,^20^,^57–59^ Although our sample included quite a large number of physicians from many different medical specialties and hierarchical levels, their representation in the different focus groups varied, and only voluntary physicians participated. This may have prevented us from grasping other physicians’ perceptions and experiences of L&M and may have limited the generalizability of our findings. However, the richness of our data lies in the fact that we included physicians from various hierarchical levels and were able to show similarities and differences in perceptions between physicians at different stages of their professional development. The percentage of fellows among all participants was high (52%). Although this over-representation may have masked perceptions of other groups of physicians, we think that it allowed to highlight similarities and differences of perceptions with other groups of physicians inside the institution. In addition, their perceptions were of special interest given their new leading positions. Finally, the time of the study may also have influenced participants’ responses. It took place a year after the beginning of the COVID-19 pandemic, during which healthcare leadership and decision-making ability were essential.^60^,^61^ This may have increased physicians’ awareness of their gaps in leadership skills and decreased their feelings of self-efficacy. However, these dimensions were not explored in our study.

## Conclusion

Physicians’ leadership skills are still mainly acquired on the job, and institutional norms do not encourage the clarification of leadership roles and processes. Given the complexity of healthcare organizations, physicians’ training in L&M skills, together with more explicit and clear institutional processes, may help improve physicians’ self-efficacy and help develop their identity as leaders.

## Data Availability

The datasets used and/or analyzed during the current study are available from the corresponding author upon reasonable request.
